# The efficacy and safety of immune checkpoint inhibitors in non-small cell lung cancer patients of different age groups: a meta-analysis

**DOI:** 10.1007/s12094-019-02241-5

**Published:** 2019-11-20

**Authors:** S.-Y. Zheng, H.-J. Cui, H. Duan, Y.-M. Peng, Q. Li, C.-Y. Sun, J.-Y. Zhang, W. Shen, X. Zhang, K. Tan, X. Jiang

**Affiliations:** 1 Beijing University of Chinese Medicine, Beijing, China; 2 Department of Integrative Oncology, China-Japan Friendship Hospital, 2 Yinghua Dongjie, Hepingli, Beijing, 100029 People’s Republic of China; 3 Fangshan Hospital, Beijing University of Chinese Medicine, Beijing, People’s Republic of China

**Keywords:** Immune checkpoint inhibitors, Non-small cell lung cancer (NSCLC), Elderly patients, Meta-analysis

## Abstract

**Background:**

Age is closely related to the efficacy of treatment for non-small cell lung cancer (NSCLC) patients. Latest clinical trials have proved the better overall survival (OS) for the use of immune checkpoint inhibitors verse chemotherapy in NSCLC patients. However, we had no clear idea of the efficacy of them in elderly patients. So we conducted a meta-analysis to compare the efficacy of immune checkpoint inhibitors for NSCLC patients of different age groups and summarized overall treatment-related adverse events.

**Materials and methods:**

PubMed, EMBASE, Web of Science and the Cochrane Library were searched for all clinical trials in NSCLC until 30th of April 2019. Eligible studies included randomized controlled trials (RCTs) comparing immune checkpoint inhibitors with chemotherapy in NSCLC patients. The hazard ratio (HRs) and 95% confidence intervals (CIs) of OS, progression-free survival or adverse events (AEs) were used.

**Results:**

A total of 4994 patients from 8 RCTs were included. Immune checkpoint inhibitors significantly prolonged the OS (HR, 0.73; 95% CI, 0.61–0.89) versus chemotherapy in NSCLC patients who were less than 65 years old. Also, they prolonged the OS (HR, 0.74; 95% CI, 0.59–0.93) in NSCLC patients who were more than 65 years old. However, there was no statistical significance of OS (HR, 0.87; 95% CI, 0.57–1.30) among NSCLC patients who were more than 75 years old. It also showed that the single use of immune checkpoint inhibitors had fewer all-grade AEs.

**Conclusion:**

Regardless of the NSCLC patients who were less or more than 65 years, immune checkpoint inhibitors could achieve better OS than chemotherapy. But there was no significant difference when NSCLC patients who were more than 75 years old. Older patient should be offered immune therapies if it is possible and the mechanism in old age treatment should be further studied.

## Introduction

Recently, with the increasing use of immune checkpoint inhibitors in large randomized controlled trials, the immune therapy has gradually turned into mainstream cancer therapy [1, 2]. The monoclonal antibodies against CTLA-4 and PD-1 have been the best studied immune therapies so far [3]. As to the squamous non-small cell lung cancer (NSCLC), Nivolumab and Pembrolizumab which are the antibodies against PD-1 have become the first-line therapy superior to chemotherapy [1, 4]. Other immune checkpoint inhibitors have also been the second-line therapy as to the advanced lung adenocarcinoma. Meanwhile, many trials which aim to prove the effectiveness of immune therapy are still under way [5]. There have been many reviews [6–8] in studying the factors which might affect the effectiveness, such as the smoking status, PD-1 expression status, tumor mutation burden (TMB), and so on.

However, very few studies have researched whether age would affect the effectiveness of immune therapy. As we all know, the immune checkpoint inhibitors attack tumor cells by activating our own immune system. Yet the elderly patients often have the problems of immunosenescence [9] and autoimmunity, which could affect their own immune system and then decrease the efficacy of immune checkpoint inhibitors. Some literatures have shown that about 50% of diagnoses in patients aged over 65 years, which illustrates that cancer is predominantly an aged disease. Also it is predicted that in the next decade, people aged over 65 will be diagnosed about 70% of new cancers [10, 11]. So it is very significant to check whether the newly developing therapy to be suitable for which age groups. Then we conducted a meta-analysis to compare the efficacy and safety of immune checkpoint inhibitors versus chemotherapy for NSCLC patients of different age groups.

## Materials and methods

### Literature search

This meta-analysis and systematic review were reported in accordance with the Preferred Reporting Items for Systematic Reviews and Meta-Analyses (PRISMA) Statement [12]. PubMed, EMBASE, Web of Science and the Cochrane Central Register of Controlled Trials (Central) databases were searched for potentially relevant studies until 30th of April 2019.

We searched studies from these databases in all fields with“Nivolumab” OR “Opdivo” OR “ONO-4538” OR “MDX-1106” OR “BMS-936558” OR “Ipilimumab” OR “Yervoy” OR “MDX-010” OR “MDX-CTLA-4” OR “Pembrolizumab” OR “Keytruda” OR “Lambrolizumab” OR “MK-3475” OR “Atezolizumab” OR “MPDL3280A” OR “Tecentriq” OR “RG-7446” OR “Durvalumab” OR “MEDI-4736” OR “Imfinzi” OR “Avelumab” OR “MSB0010718C” OR “PD-1” OR “PD-L1” OR“PD-1/PD-L1” OR “programmed cell death 1” OR “programmed cell death ligand 1” AND “Carcinoma, Non-Small Cell Lung” OR “Lung Carcinoma, Non-Small-Cell” OR “Non-Small Cell Lung Cancer” as the keywords. Articles that were not published in English were excluded.

### Selection criteria

The inclusion criteria were listed as follows: ① The trial should enroll stage IIIB or IV NSCLC patients. ② The intervention should include PD-1, PD-L1 or CTLA-4 inhibitors. ③ The control group should be treated with chemotherapy. ④ The outcome of overall survival (OS) or progression-free survival (PFS) for NSCLC patients with different age groups should be reported. ⑤ The trials should be Phase II or III randomized controlled trials (RCTs). The following excluded criteria were used: ① Studies not in English. ② The control group contained radiotherapy or targeted drugs. ③ Studies only had abstract without full text.

### Data extraction

The data weres extracted by two authors (SYZ and HD) independently. The following information was extracted from the trials: first author, year of publication, histology of lung cancer, therapeutic line, trial phase, number of patients, experimental arms, control arms, hazard ratio (HR) of PFS or OS. The third author (HJC) assessed the data and resolved the disagreement.

### Assessment of study quality and publication bias

The risk of bias was assessed according to the Cochrane Handbook for Systematic Reviews of Interventions [13], which involves assessing bias relating to random sequence generation, allocation concealment, blinding, data integrity, selective reporting of positive and/or negative findings, and other sources of bias. Among the “other sources of bias” included: (I) were there clear inclusion/exclusion criteria; (II) were the baseline data comparable; and (III) was there any conflict of interest. All the included clinical studies have been registered. The risk of bias was assessed and validated independently by three authors (SYZ, YMP and QL); the results were cross-referenced, and any disagreements were resolved by discussion with a third author (HJC).

### Statistical analysis

Stata SE 12.0 was used to conduct our systematical review, making forest plots and describe publication bias. HR and confidence interval (CI) were used as effect sizes. If the *P* value was less than 0.05, the difference between two arms had a statistical significance. The heterogeneity would be low, moderated and high, if the *I*^2^ value was less than 25%, 25–50% and over 50%, respectively. In this analysis, the null hypothesis that the studies were homogenous would be rejected if P for heterogeneity was less than 0.10 or *I*^2^ > 50%. When there was significant heterogeneity among the results of included study, the random-effects model was used to calculate summary estimate [14]. Otherwise, the summary estimate was calculated based on the fixed effects model, reported using the inverse variance method, assuming that the studies included in the meta-analysis had the same effect size.

## Results

### Search results and patients characteristics

**Fig. 1:**
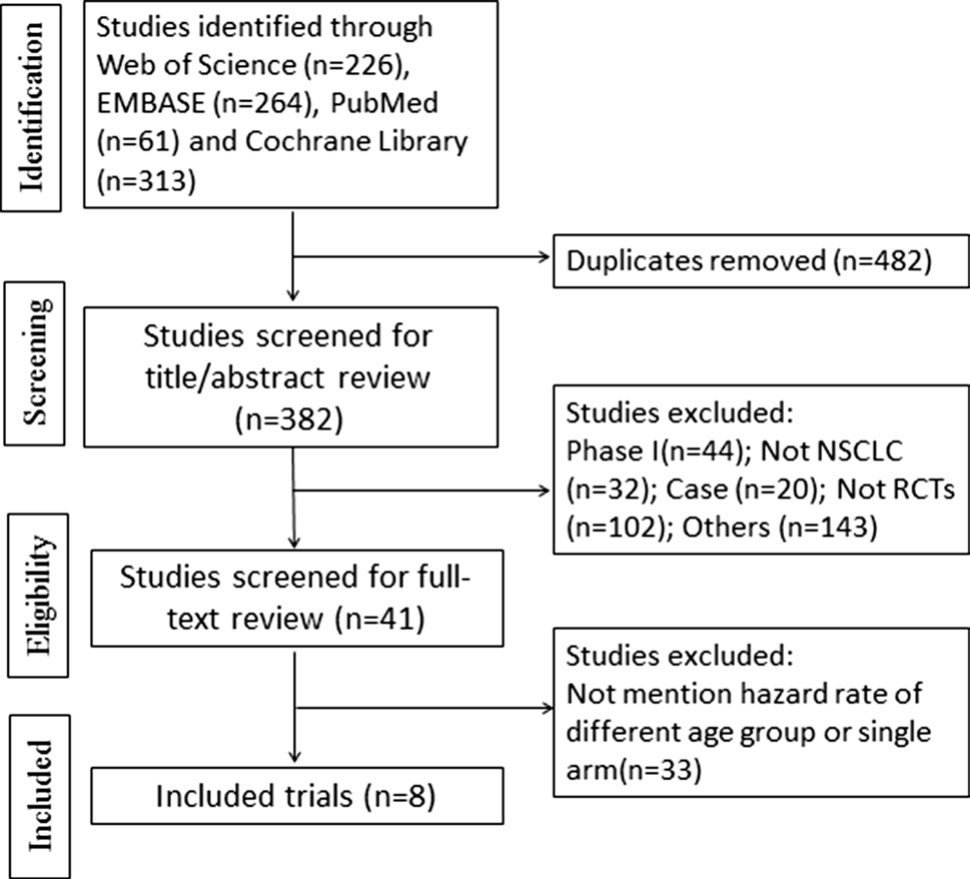
Flow diagram: selection process for the studies. Abbreviations *NSCLC* non-small cell lung cancer; *RCT* randomized controlled trial; *OS* overall survival; *PFS* progression-free survival

Of the 864 identified trials, 8 were included. The screening flow chart was shown in Fig. 1. A total of 4994 patients were enrolled in these studies [15–22]. All the trials evaluated the effectiveness of immune checkpoint inhibitors for different age groups, including 3 with pembrolizumab, 2 with Nivolumab, 1 with Atezolizumab, 1 with Ipilimumab, 1 with Avelumab. All the trials are RCTs and patients all have NSCLC. The characteristics of the trials were summarized in Table 1.

**Table 1:** The characteristics of the included studies

Author (Year)	Line	Phase	Histology	Stage	Experimental arms(s)	Immune target	Control arms(s)	Number	Follow-Up (months)	CTCAE version
Borghaei et al. [15]	Non first-line	III	Non-squamous NSCLC	IIIB or IV	Nivolumab	PD-1	Docetaxel	582	18	4.0
Reck et al. [16]	First-line	III	NSCLC	IV	Pembrolizumab	PD-1	Platinum-based chemotherapy	305	18.7	4.0
Carbone et al. [17]	First-line	III	NSCLC	IV	Nivolumab	PD-1	Platinum-based chemotherapy	541	17.4	4.0
Govindan et al. [18]	First-line	III	Squamous NSCLC	IV	Ipilimumab + Paclitaxel and Carboplatin	CTLA-4	Paclitaxel + carboplatin	749	16	4.0
Rittmeyer et al. [19]	Non first-line	III	NSCLC	IIIB or IV	Atezolizumab	PD-L1	Docetaxel	850	21	4.0
Barlesi et al. [20]	Non first-line	III	NSCLC	IIIB or IV	Avelumab	PD-L1	Docetaxel	792	18.9	4.0
Gandhi et al. [21]	First-line	II	Non-squamous NSCLC	IV	Pembrolizumab + chemotherapy	PD-1	Chemotherapy	616	20.4	4.0
Paz-Ares et al. [22]	First-line	III	Squamous NSCLC	IV	Pembrolizumab + chemotherapy	PD-1	Chemotherapy	559	19.1	4.0

### Quality of the included studies and publication bias

**Fig. 2:**
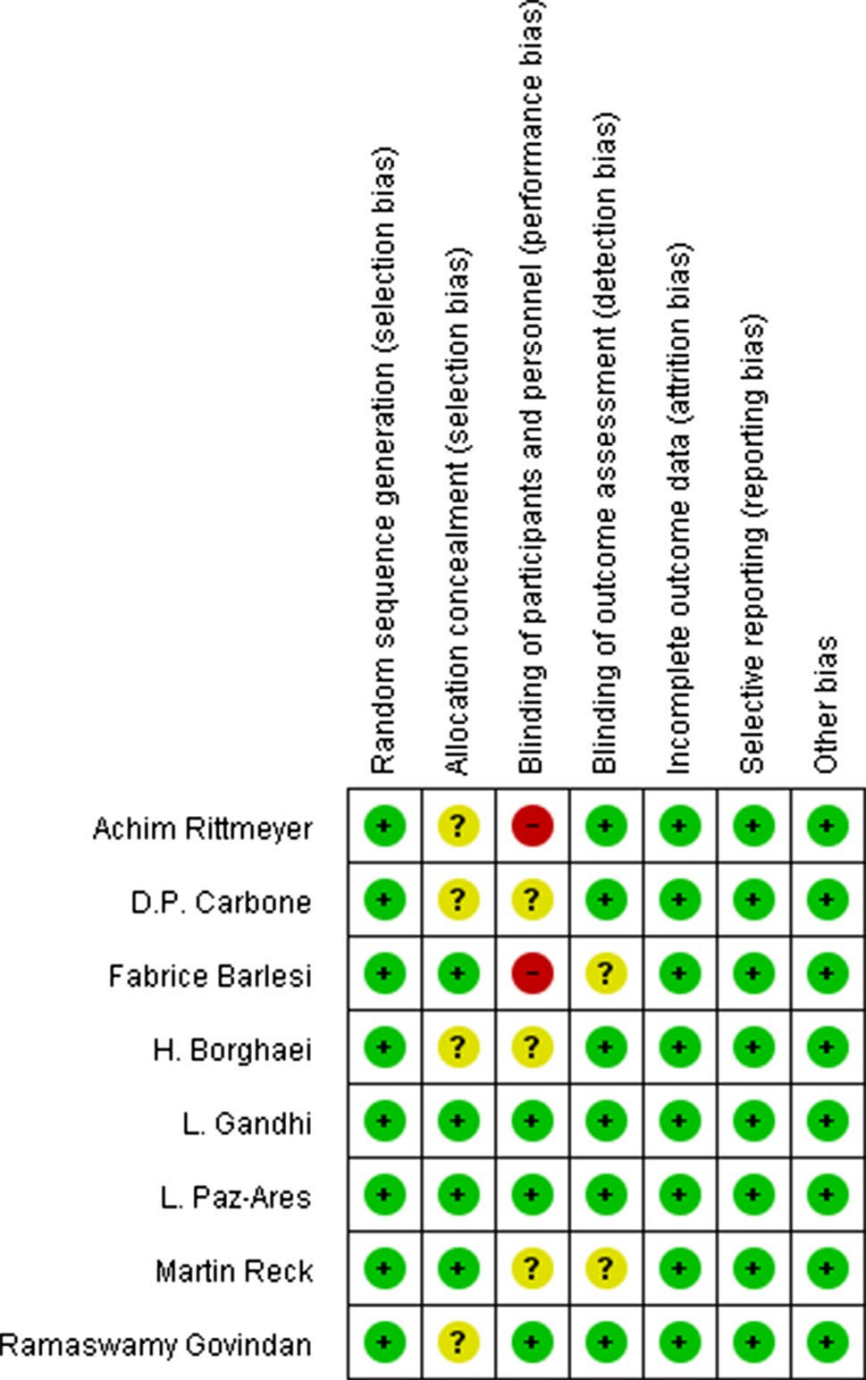
The Assessment of risk of bias

**Fig. 3:**
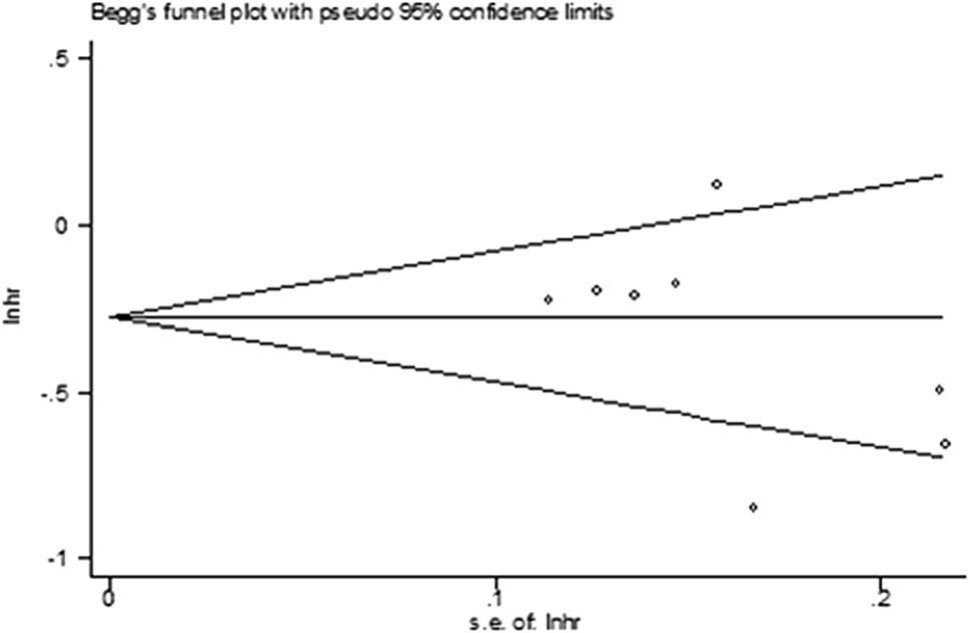
The funnel plot

Some of the included studies had a high [19, 20] or unclear risk [15–17] of performance bias, due to the difficulty in the blinding of participants and personnel. All of them had a comparatively complete report of the outcome data. The assessment of the risk of bias was shown in Fig. 2. No publication bias was observed in those studies (*P* = 0.212) by Begg’s test and the funnel plot was shown in Fig. 3.

### OS

We explored the heterogeneity via subgroup analysis based on the type of experimental arms, the class of immune target, different tumor stage and the line of therapy. And the analysis outcomes using both random-effect and fixed-effect model were shown in Tables 2 and 3.

**Table 2:** Subgroup analysis of pooled hazard ratios and 95% CI of overall survival for patients aging less than 65

Subgroup	*N*	Random-effects model		Fixed-effects model		Heterogeneity	
		HR (95% CI)	*P*	HR (95% CI)	*P*	*I*^2^ (%)	*P*
Type of experimental arms	8	0.73 (0.61, 0.89)	0.001	0.76 (0.69, 0.84)	< 0.00001	68	0.002
Immune therapy + chemotherapy	3	0.58 (0.38, 0.88)	0.01	0.63 (0.53, 0.76)	< 0.00001	79	0.008
Immune therapy	5	0.83 (0.71, 0.98)	0.03	0.84 (0.74, 0.95)	0.005	35	0.19
Immune target	8	0.73 (0.61, 0.89)	0.001	0.76 (0.69, 0.84)	< 0.00001	68	0.002
PD-1	5	0.67 (0.48, 0.94)	0.02	0.71 (0.61, 0.82)	< 0.00001	80	0.0005
PD-L1	2	0.82 (0.68, 0.97)	0.02	0.82 (0.68, 0.97)	0.02	0	0.78
CTLA-4	1	0.82 (0.64, 1.04)	0.1	0.82 (0.64, 1.04)	0.1	–	–
Tumor stage	8	0.73 (0.61, 0.89)	0.001	0.76 (0.69, 0.84)	< 0.00001	68	0.002
IIIB or IV	3	0.81 (0.70, 0.94)	0.005	0.81 (0.70, 0.94)	0.005	0	0.005
IV	5	0.67 (0.48, 0.94)	0.02	0.72 (0.62, 0.83)	< 0.00001	81	0.0004
The line of therapy	8	0.73 (0.61, 0.89)	0.001	0.76 (0.69, 0.84)	< 0.00001	68	0.002
First line	5	0.67 (0.48, 0.94)	0.02	0.72 (0.62, 0.83)	< 0.00001	81	0.0004
Subsequent line	3	0.81 (0.70, 0.94)	0.005	0.81 (0.70, 0.94)	0.005	0	0.005

**Table 3:** Subgroup analysis of pooled hazard ratios and 95% CI of overall survival for patients aging more than 65

Subgroup	*N*	Random-effects model		Fixed-effects model		Heterogeneity	
		HR (95% CI)	*P*	HR (95% CI)	*P*	*I*^2^ (%)	*P*
Type of experimental arms	6	0.74 (0.59, 0.93)	0.009	0.75 (0.66, 0.86)	< 0.0001	65	0.01
Immune therapy + chemotherapy	2	0.69 (0.53, 0.91)	0.007	0.69 (0.53, 0.91)	0.007	0	0.60
Immune therapy	4	0.76 (0.54, 1.05)	0.10	0.77 (0.66, 0.89)	0.0006	78	0.004
Immune target	6	0.74 (0.59, 0.93)	0.009	0.75 (0.66, 0.86)	< 0.0001	65	0.01
PD-1	4	0.70 (0.50, 0.99)	0.05	0.75 (0.62, 0.90)	0.002	71	0.02
PD-L1	2	0.79 (0.54, 1.16)	0.23	0.75 (0.63, 0.91)	0.003	74	0.05
Tumor stage	6	0.74 (0.59, 0.93)	0.009	0.75 (0.66, 0.86)	< 0.0001	65	0.01
IIIB or IV	2	0.79 (0.54, 1.16)	0.23	0.75 (0.63, 0.91)	0.003	74	0.05
IV	4	0.70 (0.50, 0.99)	0.05	0.75 (0.62, 0.90)	0.002	71	0.02
The line of therapy	6	0.74 (0.59, 0.93)	0.009	0.75 (0.66, 0.86)	< 0.0001	65	0.01
First line	4	0.70 (0.50, 0.99)	0.05	0.75 (0.62, 0.90)	0.002	71	0.02
Subsequent line	2	0.79 (0.54, 1.16)	0.23	0.75 (0.63, 0.91)	0.003	74	0.05

**Fig. 4:**
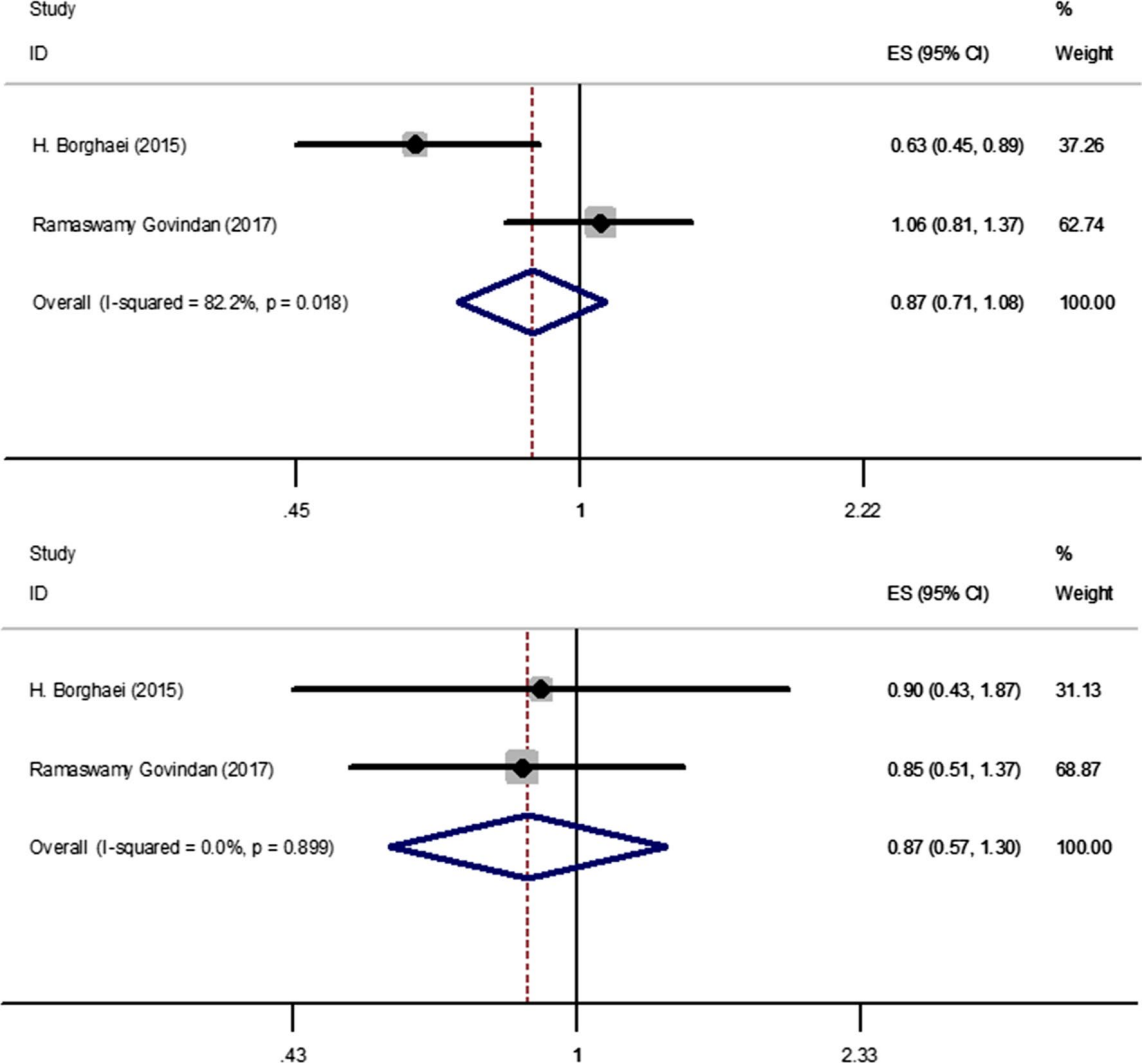
1. Forest plot for the OS of patients who were between 65 to 75 years of old. 2. Forest plot for the OS of patients who were more than 75 years old. Abbreviations *OS* overall survival

There were two trials which compared the OS [15, 18] for NSCLC patients who were between 65 and 75 years old. There was high heterogeneity in OS (*I*^2^ = 82.2%) analysis. Hence, random-effect model was used in this analysis. The meta-analysis showed (Fig. 4-1) that immune checkpoint inhibitors versus chemotherapy had no statistical significance in OS (HR, 0.87; 95%CI, 0.71–1.08).

There were two trials which compared the OS [15, 18] for NSCLC patients who were more than 75 years old. There was low heterogeneity in OS (*I*^2^ = 0%) analysis. Hence, fixed-effect model was used in this analysis. The meta-analysis showed (Fig. 4-4) that immune checkpoint inhibitors versus chemotherapy had no statistical significance in OS (HR, 0.87; 95%CI, 0.57–1.30).

### PFS

**Fig. 5:**
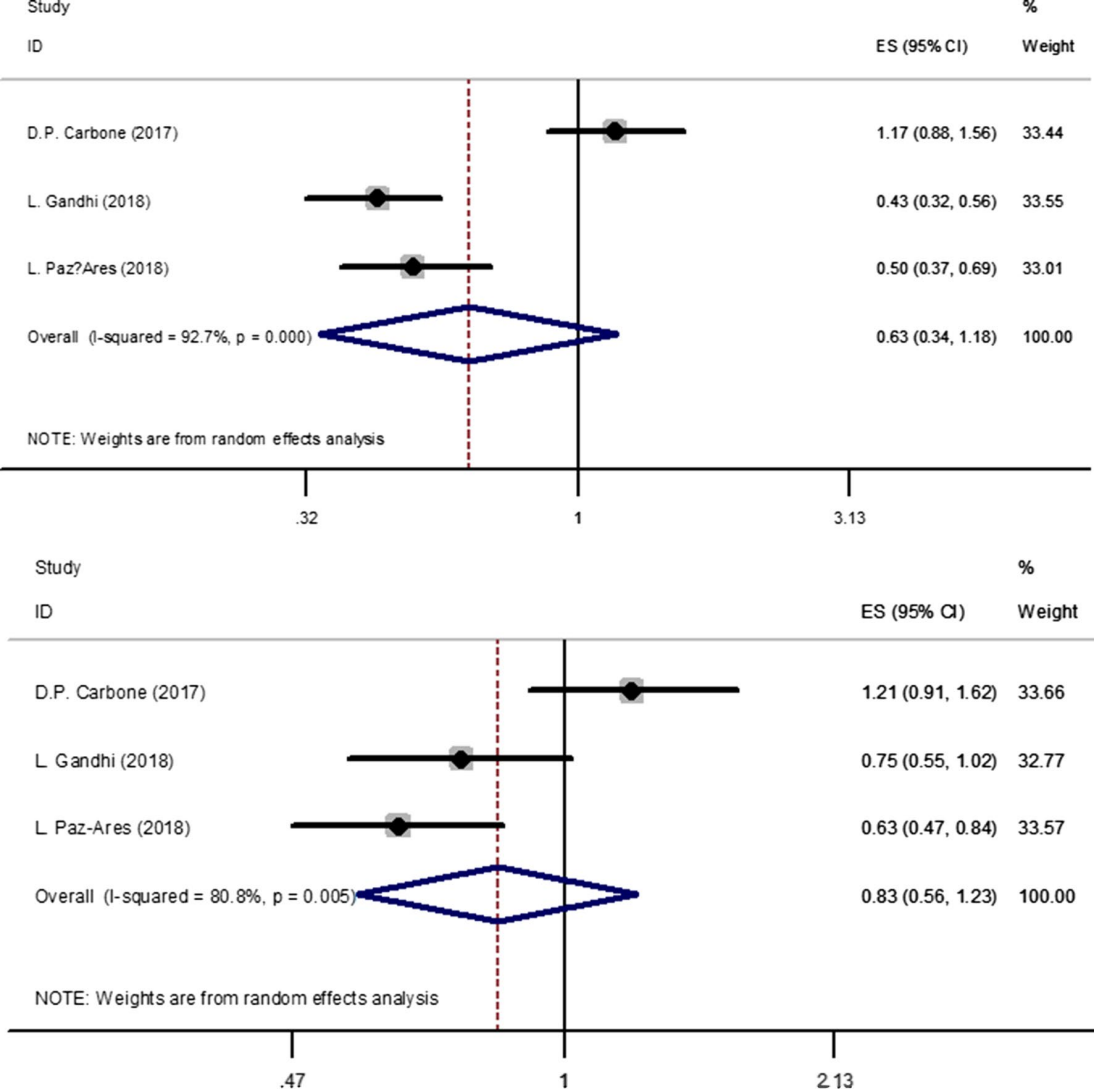
1. Forest plot for the PFS of patients who were less than 65 years old. 2. Forest plot for the PFS of patients who were more than 65 years old. Abbreviations *PFS* progression-free survival

There were three trials which compared the PFS [17, 21, 22] for NSCLC patients who were less than 65 years old. There was high heterogeneity in PFS (*I*^2^ = 92.7%) analysis. Hence, random-effect model was used in this analysis. The meta-analysis showed (Fig. 5-1) that immune checkpoint inhibitors versus chemotherapy had no statistical significance in PFS (HR, 0.63; 95%CI, 0.34–1.18).

There were four trials which compared the PFS [17, 21, 22] for NSCLC patients who were more than 65 years old. There was high heterogeneity in PFS (*I*^2^ = 80.8%) analysis. Hence, random-effect model was used in this analysis. The meta-analysis showed (Fig. 5-2) that immune checkpoint inhibitors versus chemotherapy had no statistical significance in PFS (HR, 0.83; 95%CI, 0.56–1.23).

### Adverse events

**Fig. 6:**
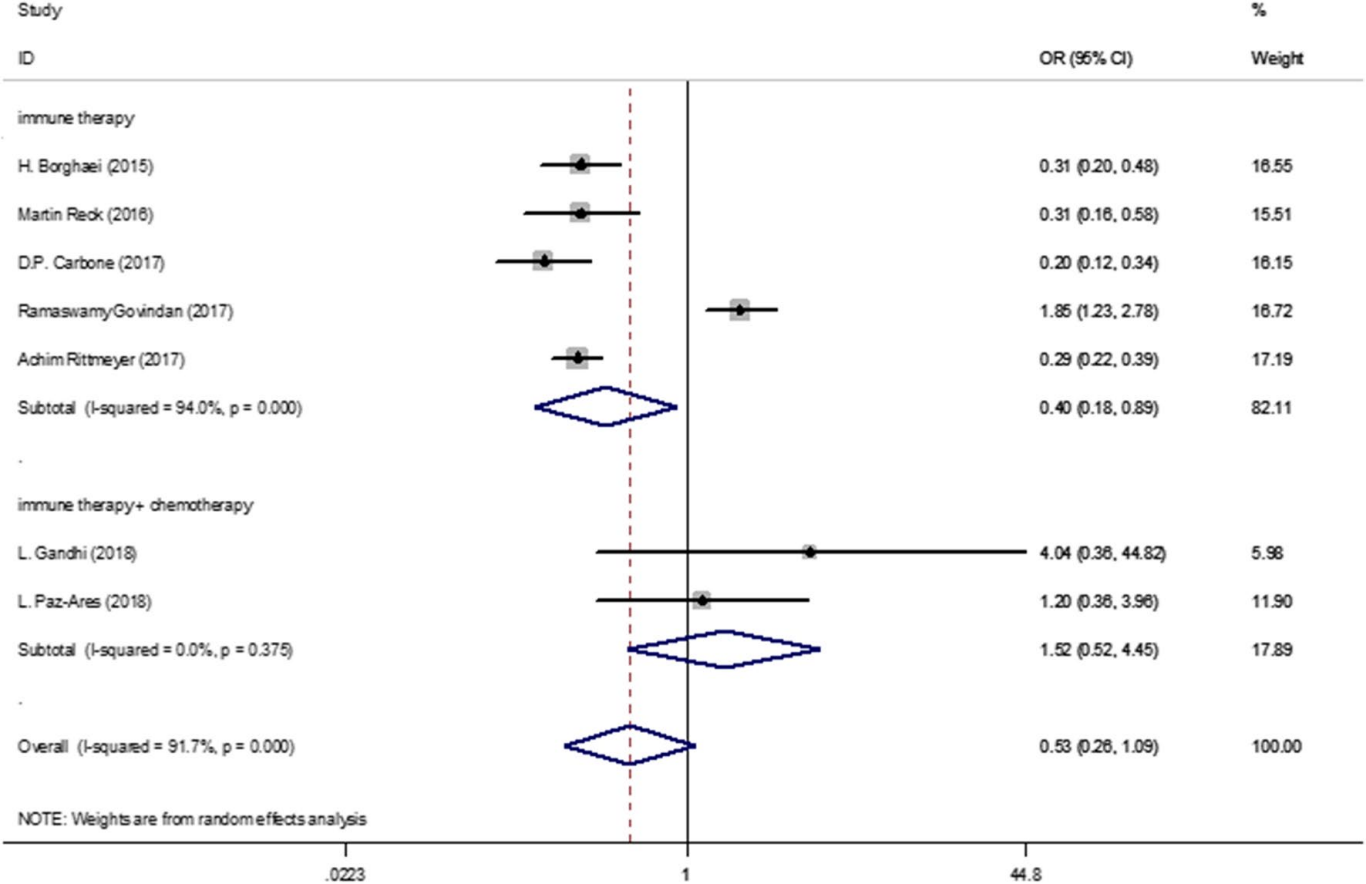
Forest plot for any AEs of patients in studies included. Abbreviations *AE* Adverse events

There were seven trials [15–19, 21, 22] which compared any adverse events for NSCLC patients. There was high heterogeneity in overall (*I*^2^ = 91.7%) analysis. Hence, random-effect model was used in this analysis. The comparison showed (Fig. 6) that the single use of immune checkpoint inhibitors had fewer all-grade AEs (RR, 0.40; 95%CI, 0.0.18-0.89; *P* = 0.000) in NSCLC patients compared with chemotherapy, while combination of immune therapy and chemotherapy had similar AEs (RR, 1.52; 95%CI, 0.52–4.45; *P* = 0.375) as chemotherapy.

## Discussion

Cancer is a disease of the elderly with more than 50% of new cases occurring in adults older than 65 years [23]. And in fact, the care of the elderly is more complicated than the young or middle age people, due to gradual organ dysfunction, slow drug reaction or many concomitant diseases. So it is significant to research whether the immune therapy could have similar advantage over chemotherapy in different age groups.

Our study included 4994 patients from 8 RCTs (3 with pembrolizumab; 2 with Nivolumab; 1 with Atezolizumab; 1 with Ipilimumab; 1 with Avelumab), covering the immune checkpoint inhibitors used in more than phase II clinical trials. Based on the data provided by the included trials, we classified the patients into different subgroups, which were less than 65 years old, over 65 years old, from 65 to 75 years old and over 75 years old. The meta-analysis showed that no matter immune therapy or the combination of immune therapy and chemotherapy both prolonged the OS (HR, 0.73; 95% CI, 0.61–0.89) versus chemotherapy in NSCLC patients who were less than 65 years old. And to explore the heterogeneity, we made detailed subgroup analyses which showed all the subgroups other than the immune target (CTLA-4) achieved a better OS than chemotherapy. Also, they prolonged the total OS (HR, 0.74; 95% CI, 0.59–0.93) compared with chemotherapy in NSCLC patients who were more than 65 years old. The result is consistent with the observational study [24] in Italy, which also showed that the elderly patients could benefit from the immune therapy like younger patients.

However, when we made detailed subgroup analyses of the data from patients aging more than 65, we found there were no differences as to the subgroups of using immune therapy alone, the immune target being PD-L1, tumor stage IIIB or IV, the using in the subsequent line of therapy. And there was no statistical significance of OS (HR, 0.87; 95% CI, 0.57–1.30) in the comparison among NSCLC patients who were more than 75 years old, which was similar to the results in one cohort [25]. It is worth noting that there was also no statistical significance of PFS (HR, 0.81; 95%CI, 0.61–1.08) in the comparison among NSCLC patients who were less or more than 65 years old and OS (HR, 0.83; 95%CI, 0.50–1.38) among patients from 65 to 75 years old (but there were only two studies reporting the data, so the results could not be carefully considered). To some extent, it illustrated that the effect of immunotherapy gradually declined with age. And the combination of immune therapy and chemotherapy or using immune therapy in the first line may still have a better response than immune therapy alone or using in the subsequent lines in the older patients compared with chemotherapy. And as we all know the advantage of immune therapy is once it works it will have lasting effects, so the golden standard to describe the efficacy of immune therapy is absolutely OS.

Cytotoxic T-lymphocyte-associated antigen 4 (CTLA-4) and programmed cell death protein-1 (PD-1) are the two most well studied immune regulatory checkpoint pathways in cancer [26]. So many drugs have been investigated through this mechanism, such as Ipilimumab which is the antibody against CTLA-4, Nivolumab and Pembrolizumab which are the antibodies against PD-1, and so on [27, 28]. These drugs do not attack tumor cells like chemotherapeutic drugs but regulate the molecules around T lymphocytes which help human immune system to recognize cancers. In essence, these drugs do not aim to activate the immune system, but rather to remove the immune escape caused by tumor cells [29]. Then T lymphocytes could attack the tumor invaders normally as they attack pathogens. As to the mechanism, the efficacy of immune therapy is closely related to the human immune system itself.

It is known that an age-related immune dysfunction, which is often called immunosenescence, may influence the anti-tumor effect of these drugs [30]. It has been proved that the major factor involved in the pathophysiology of immunosenescence is the diminished T-cell mediated immunity [31]. Yet the immune checkpoint inhibitors work by removing the immune escape of tumor cells from T lymphocytes. And the meta-analysis showed that the effect of immunotherapy definitely declined with age. So it is speculated that the decreased effectiveness might be related to the immunosenescence in old age. In addition, age remains an important risk factor for autoimmunity, either with clinical impact or with only biological modification [32]. This may be connected with the clinical fact that elderly people are more likely to receive other treatments before immunotherapy. Other treatments especially cytotoxic drugs might influence the normal function of immune system. This explains the results, why using immune therapy in the first line may still have a better response in the older patients. And we can infer that the key to the efficacy of the immune therapy is more likely to be related to the treatment histories. And the theory can simultaneously explain why the effect of immunotherapy gradually declined with age in most cases.

Although we strictly followed the PRISMA statement, our study still had several potential limitations. First, some of the included studies had a high [19, 20] or unclear risk [15–17] of performance bias, by assessing the quality of the included studies. This was due to the difficulty in the blinding of participants and personnel. Also some trials [15, 17–19] might have some selection bias due to their unclear report of allocation concealment. Therefore, except for the OS analysis of NSCLC patients over 75 years old, other analyses have higher heterogeneity. Second, from the funnel plot in our study, there might be a little publication bias (two points outside the funnel plot) in this meta-analysis. Third, there were only two trials that included the NSCLC patients who were between 65 and 75 years old and more than 75 years old, so their results of meta-analysis could be for reference only. Fourth, nearly all the included trials limited the patients within an Eastern Cooperative Oncology Group (ECOG) performance-status score of 0 or 1. While the elderly patients were more likely to have poor physical status, so part of data from the elderly might not be reported. So it is necessary to conduct more and more real-world researches to test the efficacy of them in the elderly patients with different ECOG scores. Last but not least, all the included randomized control trials did not report the occurrence of adverse events in different age groups, so we only reported the overall comparison of AEs for readers’ reference. So there was no way to make a meta-analysis of checking the safety of immune checkpoint inhibitors in the elderly patients. And there were only a few retrospective researches [24, 25] that reported the adverse events in different age groups. So it is necessary to complementally report the adverse events in different age groups in those large randomized control trials. If possible, further studies need to include these patients who were more than 75 years old. Also the mechanism of immunosenescence and autoimmunity in old age groups should be further studied.

## Conclusion

In conclusion, regardless of the NSCLC patients who were less or more than 65 years old, immune checkpoint inhibitors could achieve better OS than chemotherapy. But there was no significant difference when NSCLC patients who were more than 75 years old. So if the patients were older than 75, they should weight the advantages and disadvantages for the best therapeutic schedule. Older patient should be offered immune therapies if it is possible and the mechanism of treating the elderly with them should be further studied.
